# Towards rational computational peptide design

**DOI:** 10.3389/fbinf.2022.1046493

**Published:** 2022-10-21

**Authors:** Liwei Chang, Arup Mondal, Alberto Perez

**Affiliations:** ^1^ Department of Chemistry, University of Florida, Gainesville, FL, United States; ^2^ Quantum Theory Project, University of Florida, Gainesville, FL, United States

**Keywords:** peptide, computational modeling, structure prediction, peptide-protein interactions, peptide self-assembly

## Abstract

Peptides are prevalent in biology, mediating as many as 40% of protein-protein interactions, and involved in other cellular functions such as transport and signaling. Their ability to bind with high specificity make them promising therapeutical agents with intermediate properties between small molecules and large biologics. Beyond their biological role, peptides can be programmed to self-assembly, and they are already being used for functions as diverse as oligonuclotide delivery, tissue regeneration or as drugs. However, the transient nature of their interactions has limited the number of structures and knowledge of binding affinities available–and their flexible nature has limited the success of computational pipelines that predict the structures and affinities of these molecules. Fortunately, recent advances in experimental and computational pipelines are creating new opportunities for this field. We are starting to see promising predictions of complex structures, thermodynamic and kinetic properties. We believe in the following years this will lead to robust rational peptide design pipelines with success similar to those applied for small molecule drug discovery.

## Introduction

Computational modeling is routinely used in the early stages of the drug discovery process to identify molecules that might bind with high affinity to a particular protein receptor. Three key aspects that contribute to the success of computational tools are: 1) the availability of small molecule virtual libraries (e.g., (Irwin and Shoichet, 2005; Shivanyuk et al., 2007; Bento et al., 2014)), 2) the efficiency of docking software to identify candidates from the virtual libraries (Murugan et al., 2022), and 3) the accuracy of physics-based approaches such as alchemical free energy perturbation to determine relative and absolute binding affinities amongst candidate molecules binding a receptor (Bhati et al., 2017; Fratev and Sirimulla, 2019; Lee et al., 2020; Limongelli, 2020; Mey et al., 2020). Some of the limitations include the presence of multiple binding modes, plasticity in the receptor, large conformational changes in the binding molecule, highly charged systems, and comparison across different series of compounds (Chodera et al., 2011; Mobley and Klimovich, 2012; Mey et al., 2020). Despite the progress of computational platforms for drug discovery, small molecule drugs typically require a pocket in the receptor protein in order to bind (Laskowski et al., 1996; Liang et al., 1998; Gao and Skolnick, 2013).

**FIGURE 1:**
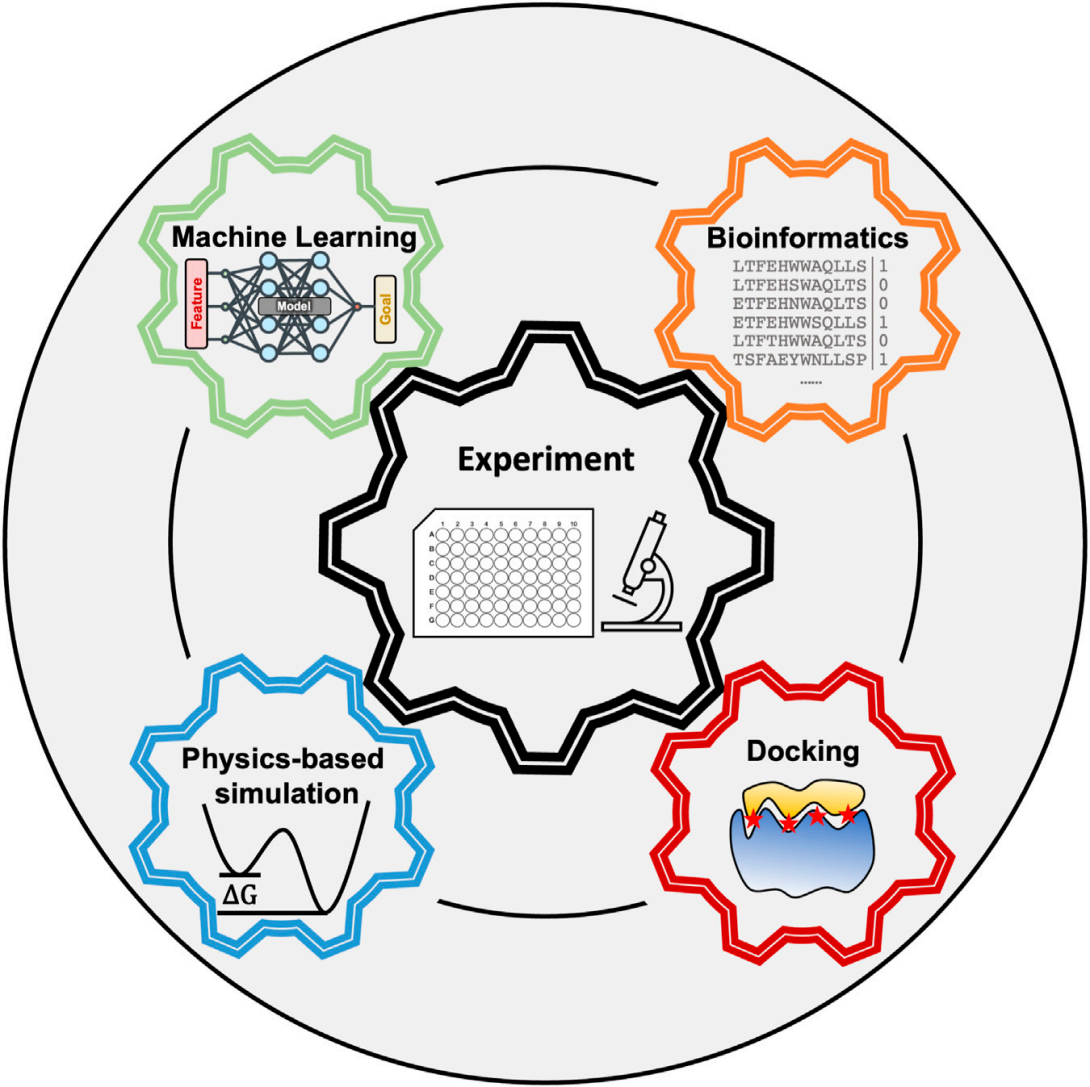
Peptide modeling requires synergy between multiple computational techniques and experiments.

Peptides are flexible molecules that can bind specifically to receptors even in the absence of pockets–targets that were once deemed undruggable by small molecule drugs (Balliu and Baltzer, 2017; Wang et al., 2021). Our cells already use peptides as signaling molecules (Cunha et al., 2008; Foster et al., 2019; Dubas et al., 2021), and many protein-protein interactions take place through peptide epitopes (Milroy et al., 2014; Pelay-Gimeno et al., 2015; Wai et al., 2018; Aiyer et al., 2021). Thus, peptides offer the possibility to inhibit interactions present in disease pathways (Pelay-Gimeno et al., 2015). Furthermore, peptides are highly programmable for self-assembly, allowing the generation of functionalized fibers with applications ranging from scaffolds for tissue regeneration (Loo et al., 2015a) to bioink (Loo et al., 2015b). Successful computational pipelines would allow the rational identification of peptides that bind with high affinity to a particular receptor or have specific self-assembly properties. However, peptide’s flexible nature, the large sequence space, and presence of multiple weak interactions that stabilize the complex, have stymied the development of peptide discovery pipelines. Achieving success in modeling peptide complexes will require synergy between several fields which we discuss in this perspective (see Figure 1).

Infrastructure similar to small molecule discovery pipelines are already in place for peptides, with an overall lower success rate. Instead of small molecule virtual libraries, bioinformatic approaches are typically used to derive peptide libraries based on known information from the receptor and known binding partners, reducing the active sequence space from millions (20^n^, where *n* is the length of the peptide) to thousands of sequences. But, dealing with peptide flexibility reduces the efficiency of both docking tools and scoring functions to predict accurate structures (Rentzsch and Renard, 2015; Wang et al., 2016; Ciemny et al., 2018). Surprisingly, a recent implementation of AlphaFold (AF) for peptide docking showed an unprecedented success–despite being trained for a different task (protein structure prediction) (Jumper et al., 2021; Ko and Lee, 2021; Tsaban et al., 2022). Combining search strategies and template-based strategies, PatchMAN has recently surpassed even the successes from AF under certain scenarios, leading the way into the structural characterization of previously unknown peptide-protein interactions (Khramushin et al., 2022).

**FIGURE 2:**
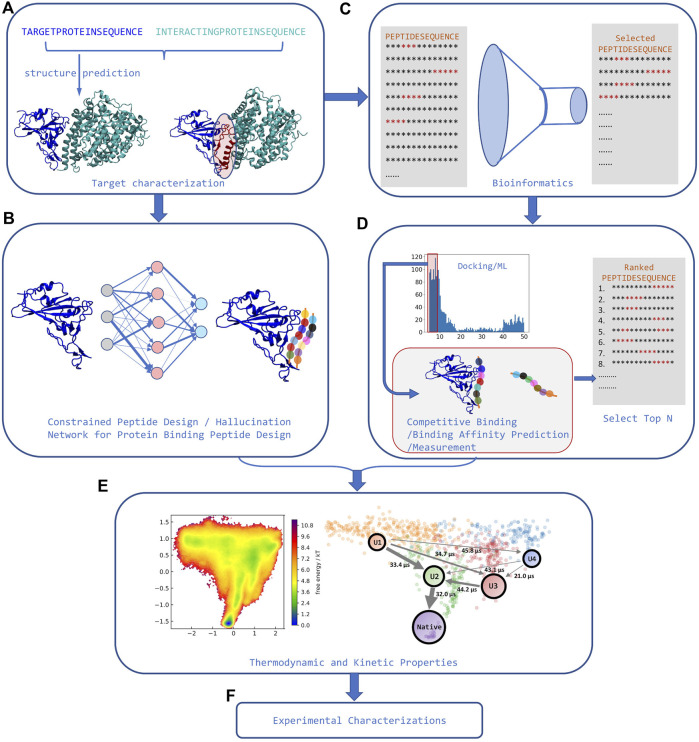
Strategies for rational peptide design. **(A)**. The structure of target protein and its interacting partners is first identified, and interface features are extracted from the complex. **(B)** Machine learning models can use these interface features to perform constrained peptide design for that target. **(C)** Alternatively, using these interface features, bioinformatics tools can significantly narrow down the sequence search space. **(D)** Peptide docking tools or AI tools can then be used to predict binding in the now reduced set of candidate sequences. Competitive binding study or high throughput binding affinity measurement can rank order the selected sequences from the docking step. **(E)** In some cases, orthogonal modeling capable of predicting kinetic and/or thermodynamic properties can further narrow down the number of possible sequences selected for experimental determination (adapted from (Chang and Perez, 2022b)). **(F)** Finally, experimental characterization and validation can be carried out for the now manageable number of predicted peptide binders.

The last step needed for a pipeline that can identify high affinity peptide binders is a way to rank-order peptides by binding affinity that overcomes limitations in traditional scoring functions (see Figure 2). A recent assay using competitive binding with AF has shown the potential to differentiate between weak and strong peptide binders under certain conditions (Chang and Perez, 2022a). Some of the limitations observed in the assay are similar to those found when using AF for docking and stem from the fact that AF was only parametrized for predicting protein structures, rather than binding–thus AF has an implicit bias against bound peptides. A different approach using an extra layer on top of AF trained on binding affinity data for the Major Histocompatibility Complex (MHC type I and II) shows the ability to differentiate active binders from inactive peptides (Motmaen et al., 2022). Finally, some physics-based approaches try to establish methodologies capable of capturing both the flexibility and binding affinity based on known binding modes (Morrone et al., 2017; Liu et al., 2020; Mondal et al., 2022). However, they are still more computationally demanding and there are no good benchmark sets showing transferability, convergence, and associated errors.

Based on our current understanding of the field we expect significant changes coming from better training of AF like strategies to the problem of peptide-protein complexes. There are already instances of this, and the community has developed efforts for facilitating the use (https://colab.research.google.com/github/sokrypton/ColabFold/blob/main/AlphaFold2.ipynb) (Mirdita et al., 2022) and training (https://github.com/aqlaboratory/openfold) of these technologies. However, these are all focused on the structure prediction. What will we need to predict binding affinities or binding mechanisms? Approaches such as the one presented for MHC are only possible when enough existing data on binding affinities is known and are thus not directly transferable to all systems. Similar to the Protein Structure Initiative (Montelione, 2012), will the collection and creation of databases of binding affinities for multiple systems (e.g., similar to those curated for transcription factor protein-DNA interactions (Chiu et al., 2019; Fornes et al., 2019)) lead to new possibilities for the virtual screening of peptide libraries based on artificial intelligence? And what is the role of physics-based approaches? Worldwide competition events such as CAPRI (Janin et al., 2003) and CASP(Moult, 2005) have spurred development, independent assessment and standardized benchmark sets for the community–and they play an important role for the development of similar model challenges in related fields (e.g. the CyroEM model challenge) (Lawson et al., 2021).

## Key areas of synergy for modeling peptide behavior

### Increasing the size of peptide-protein databases

Machine learning has already shown promising results for predicting peptide-protein interactions despite being trained for a different task–training for peptide binding will require increasing our structural and energetic understanding of these interactions. However, the transient nature of peptide-protein interactions has made it challenging to accumulate large datasets, both in terms of structures and binding free energies. Both are needed to develop computational software that predicts high affinity peptide binders (Cunningham et al., 2020; Motmaen et al., 2022). Two advances will play key roles to increase database knowledge to feed machine learning databases: 1) curating protein-protein structural databases to identify peptide epitopes and their interaction patterns, as recently shown by PatchMAN (Khramushin et al., 2022) and others (Peterson et al., 2017; Aderinwale et al., 2020), 2) establishment of high throughput/high sensitivity techniques for determining binding affinities (Nguyen et al., 2019).

The protein data bank (Berman et al., 2002) has an overrepresentation of structures amenable to the techniques needed to solve their structures. Thus, stable protein monomers are overrepresented with respect to multimeric structures. Furthermore, many intrinsically disordered proteins (IDP) contain short linear motifs (SLiMs) that bind proteins transiently through peptide epitopes 3–12 amino acids long (Krystkowiak and Davey, 2017; Ivarsson and Jemth, 2019). These interactions are hard to characterize both experimentally and computationally, but their prevalence (estimated to be up to 100,000 SLiMs) and biological importance makes their characterization especially important (Tompa et al., 2014). As many peptide sequences will be able to bind in sites were SLiMs bind, it will be more important to characterize not only binding affinities (in terms of K_d_) but also the specificity of the binding region.

A similar issue of binding affinity/specificities can be seen in determining where in a genome a transcription binding protein will bind. This community has developed high-throughput techniques to identify binding affinities both *in vivo* and *in vitro* which are summarized in position binding motifs (PBMs) and compiled in databases such as TRANSFAC(Matys et al., 2006) or JASPAR(Fornes et al., 2019). We expect to see expansion of peptide databases to the levels seen in the protein-DNA field. Many high-throughput techniques for detecting binding affinities (Tonikian et al., 2008; Ivarsson et al., 2014; Jensen et al., 2018; Parker et al., 2019) are limited by one or more of the following: low binding affinities, aggregation, rapid equilibrium, low accuracy in estimating purity and/or concentration of the peptides, qualitative (presence of binding) instead of quantitative (K_d_), and fast dissociation rates (Nguyen et al., 2019)—thus, resulting in high uncertainties. Generally, higher accuracy data requires lower throughput methods such as isothermal titration calorimetry or surface plasmon resonance (Abraham et al., 2005), limiting the amount of available data. More recently, developments such as microfluidic-based approaches (Vincentelli et al., 2015; Nguyen et al., 2019; Hein et al., 2020) enable a faster complementary method to high-throughput methods and will play a key role in increasing our understanding of peptide-protein binding affinities.

### Bioinformatics

While some high-throughput methods use combinatoric peptides libraries, bioinformatic tools use knowledge of the system to create libraries that are more likely to contain peptides that bind a particular target (Turk and Cantley, 2003). Thanks to advances in sequencing and metagenomic approaches, there are now extensive sequence libraries (Bateman et al., 2017; Breitwieser et al., 2017) and efficient tools to process them (Finn et al., 2011; Remmert et al., 2012; Potter et al., 2018). Furthermore, their importance for both computational and experimental researchers has led to effective pipelines without computational expertise requirement (Joshi and Blankenberg, 2022). Thus, bioinformatic approaches take the role of narrowing sequence space.

### Machine learning

With the rapid evolution of this field, any prediction of how exactly this field will evolve and which databases will be needed for training will likely be outdated in a few weeks. A promising trend highlighted by the application of AlphaFold to problems of protein-protein and peptide-protein structure predictions is the potential for transferability across macromolecular interactions. Recently, AlphaFold principles have been used to derive a protein-nucleic acid structure prediction software (Baek et al., 2022). Future AI should be aiming to understand macromolecular interactions independently of the type of molecule: rather than having an AI for proteins and a different for small molecules or nucleic acids we need integrated AI to handle all aspects of molecular recognition–transferable to other biomolecules like peptoids, modified proteins, nucleic acids and capable of interacting with small molecues. A second area of development for AI will be the identification and correction of biases arising from training datasets. For example, the training for finding folded states creates a bias to favor bound structures over unbound ones–predicting some protein-peptide complexes even when the peptide should not bind. Finally, the prediction of structures and binding affinities seem to be on two independent tracks at the moment–some tools are good for sampling bound states and others for rank-ordering them by binding affinity (Cunningham et al., 2020; Chang and Perez, 2022a; Motmaen et al., 2022). While development in affinity prediction has lagged behind the structure prediction problem, there is a recent increase in the number of methods available, some of them now available as webservers with promising accuracy (Romero-Molina et al., 2022). It is feasible to think that as binding affinity databases increase in size and accuracy, these two independent pieces will be trained together. In this direction, machine learning based approaches that predict peptide-protein interactions and peptide binding residues (Lei et al., 2021) can help increase or build peptide-protein databases.

### Physics-based approaches

The promise of these approaches has been their potential to capture bound states, as well as the thermodynamic and kinetic properties connecting the different states. This promise has been compromised by inaccuracies in physics models (force fields), efficient sampling strategies, and computational cost (Durrant and McCammon, 2011; Petrov and Zagrovic, 2014). At this point it seems unfeasible that physics-based approaches will be able to match the speed and accuracy of structure predictions coming from machine learning in the near-future. However, once the structure is known, it might still play a role in determining thermodynamic (e.g., K_d_) and kinetic properties (e.g., k_on_ and k_off_) (Zuckerman and Chong, 2016; Paul et al., 2021, 2017; Dickson et al., 2017; Zimmerman et al., 2018; Wang and Miao, 2020).

With an increasing number of methods that capture thermodynamics, the question is whether or not they will become computationally feasible and routine to match the success of FEP calculations in small molecules. These types of methods could address some of the limitations that are prevalent in small chemical changes (single point mutations) and explicitly account for posttranslational modifications (Edwards et al., 2021). Furthermore, the development and maturity of platforms to analyze ensembles for kinetic properties (e.g., weighted ensemble methods, and Markov state models) are leading to pioneering works capturing not only the thermodynamics but also kinetics of macromolecular interactions (Zuckerman and Chong, 2016; Zhou et al., 2017). Currently dissociation rates seem to have the largest uncertainties (by a few orders of magnitude), which can be in part explained by force fields that tend to favor compact structures. As these methods mature and force field development efforts continue to emphasize capturing folded, IDP, bound and unbound behavior, we expect the accuracy to improve.

### Design principles

Recent protein design work is already leveraging machine learning to hallucinate novel folds based on the idealized version of proteins learnt by the algorithms (Anishchenko et al., 2021; Wang et al., 2022). Furthermore, the promise of constraint design based on a given backbone structure is promising for the design of proteins and peptides that bind a particular target (Moffat et al., 2021; Anand et al., 2022). This has already led to the design of mini proteins through the combination of computation (Rosetta) and experiments (Cao et al., 2022). Recent approaches started to take physical property such as solubility of designed peptides into consideration (Kosugi and Ohue, 2022). With the maturity of this field at the hands of a few expert groups, comes the possibility to design molecules with an increasing range of properties, such as peptide macrocycles that are not only able to adopt a particular structure, but also are able to cross membranes.

### Peptide self-assembly

Peptides have programmable self-assembly properties, while maintaining the ability to incorporate functional motifs recognized by other macromolecules. Fortunately, due to these properties, peptides are increasingly used for biomaterial design applications (e.g., functionalized hydrogel scaffolds that induce cellular behavior) (Loo et al., 2015a). Despite its promise, atomistic tools to predict the structures, properties, accessibility of functional sites and contributing to the development of materials have lagged their experimental counterpart (Rauscher and Pomès, 2017; Smadbeck et al., 2014). We believe two independent directions will benefit from recent approaches: 1) the identification of new functional motifs to queue cellular behavior; 2) the ability to predict structures and physical properties of self-assembling peptides to complement experimental efforts.

### Post translational modifications

The recent Sars-COV2 pandemic highlighted the need to account for post translational modifications (PTM). Yet, as modelers we are often faced with the challenge of knowing which and where these modifications occur, lack of parameters or datasets (machine learning) to model these modifications. This issue becomes accentuated for the transient interactions of interest in peptide-protein interactions. However, the community has shown a fast response and adaptation to model these systems. We believe going forward the community will collect more data on such PTM that will improve modeling efforts. For example, many of the peptide-protein experimental binding affinity assays already incorporate the ability to introduce such PTMs (Nguyen et al., 2019)—and computational pipelines (Khoury et al., 2013; Croitoru et al., 2021) are adapting to facilitate incorporating such modifications to systems of interest.

## Discussion

We are excited by the role that recent advances in a variety of areas can have in the field of peptide-protein structure and affinity prediction. Peptides can be of interest as single molecule therapeutics or as programmable molecules that aggregate to specific patterns creating scaffolds (Perez et al., 2021). In both scenarios a functional motif will allow these peptides to target and inhibit or enhance interactions and pathways. Peptides have some intrinsic limitations like fast degradation and difficulty crossing barriers that might make them precursors of other molecules such as modified peptides (Szabó et al., 2022), mini-proteins (Cao et al., 2022) and small molecule peptidomimetics (Rubin et al., 2018; Perez, 2021). However, predicting the initial structure and identifying high affinity binders will be critical to develop these peptide derivatives. As the field moves forward, identifying novel binding motifs and the sequence/structure relationships that allows these peptides to bind will in turn increase our understanding of binding plasticity, the role of multiple binding modes in binding affinities for more accurate predictions.

A particular field of interest where we see methods expanding their presence is in material bioengineering (Pérez et al., 2013). Here, peptide epitopes embedded in fibers that make up the extra cellular matrix (ECM) bind different integrin proteins on the cellular membrane which trigger different cascade of events that govern cellular behavior (such as adhesion, growth, or migration) (Collier et al., 2010; Wade and Burdick, 2012; Loo et al., 2015a; Hellmund and Koksch, 2019). We are aware of under 100 linear motifs that bind integrins, where both the sequence and conformation are important. However, it is likely that more motifs can bind in these sites, offering opportunities for designed biomaterials. The second challenge such materials must meet is the ability to self-assemble in order to mimic the ECM–with different biomechanical properties in different tissues (e.g., softer for tissues like the brain, and stiffer for tissues like bone or muscles) (Collier et al., 2010). Computational tools in this area are scarce (Smadbeck et al., 2014; Rauscher and Pomès, 2017), but increase in computational power, force fields that better represent the balance between folded/unfolded and new sampling strategies make us optimistic in this area as well. Starting from peptides that are experimentally known to self-assemble, the first goal is to robustly and reproducible generate atomistic models of the resulting scaffold. Analysis of these scaffolds will allow us to distinguish mechanical properties like stiffness and accessibility of the functional motifs. A second requirement will be to identify pipelines that are sensitive to sequence properties: many experimental designs show the evolution of the peptide self-assembling motif, from initial sequences that barely oligomerized to successful scaffolds (Papapostolou et al., 2007). With self-assembling materials small errors in force field or machine learning preferences get amplified throughout the scaffold. Thus, a higher level of sensitivity is required. Finally, these models should be tested for functionality: are the binding motifs readily available for interaction with desired proteins (e.g., integrins that control cellular behavior). Can these models explain some of the behavior observed when mixing two successful binding motifs that have different functionality (e.g., adhesion and growth signals) (Liu et al., 2015).

## Conclusion

The rapid pace of advances in AI, coupled with increased computational power, access to larger and more curated databases and improvements in other computational modeling pipelines is changing many aspects of structural biology pipelines. We believe this presents an opportunity to advance our understanding of systems involving peptide molecules, where advances both experimental and computational have faced the challenges of working with such flexible molecules. Peptides already have multiple applications ranging from drugs, to nanodevices, or tissue regeneration to name a few. Developing robust computational pipelines to design and control the properties of these molecules will allow a rapid increase in the number of applications and uses of these molecules.

## Data Availability

The original contributions presented in the study are included in the article/supplementary material, further inquiries can be directed to the corresponding author.
